# Protocol for CLASSIC PBB: comparison of lower airway sampling strategies in children with protracted bacterial bronchitis

**DOI:** 10.1136/bmjpo-2022-001722

**Published:** 2022-11-21

**Authors:** Francis J Gilchrist, Mathew Aspey, Robert Bowler, Malcolm Brodlie, Seema Desai, Caroline Harris, Emily Hinton, Hemant Kulkarni, Aviva Ogbolosingha, Ian Sinha, Ivonne Solis-Trapala, Joanne Stock, William D Carroll

**Affiliations:** 1 Institute of Applied Clinical Science, Keele University, Keele, UK; 2 Paediatric Respiratory Services, University Hospitals of North Midlands NHS Trust, Stoke-on-Trent, UK; 3 Research and Innovation Directorate, University Hospitals of North Midlands NHS Trust, Stoke-on-Trent, UK; 4 Institute of Celular Medicine, Newcastle University, Newcastle upon Tyne, UK; 5 Paediatric Respiratory Medicine, Great North Children's Hospital, Newcastle Upon Tyne, UK; 6 Department of Microbiology, University Hospitals of North Midlands NHS Trust, Stoke-on-Trent, UK; 7 On Behalf of Patient and Public Involvement and Engagement for CLASSIC PBB, University Hospitals of North Midlands NHS Trust, Stoke-on-Trent, UK; 8 Sheffield Children's Hosptial, Sheffield Teaching Hospitals NHS Foundation Trust, Sheffield, UK; 9 Department of Respiratory Medicine, Alder Hey Children's NHS Foundation Trust, Liverpool, UK; 10 School of Medicine, Keele University, Keele, UK

**Keywords:** Microbiology

## Abstract

**Background:**

Protracted bacterial bronchitis (PBB) is an endobronchial infection and a the most common cause of chronic wet cough in young children. It is treated with antibiotics, which can only be targeted if the causative organism is known. As most affected children do not expectorate sputum, lower airway samples can only be obtained by bronchoalveolar lavage (BAL) samples taken during flexible bronchoscopy (FB-BAL). This is invasive and is therefore reserved for children with severe or relapsing cases. Most children with PBB are treated empirically with broad spectrum antibiotics. CLASSIC PBB will compare the pathogen yield from two less invasive strategies with that from FB-BAL to see if they are comparable.

**Methods:**

131 children with PBB from four UK centres referred FB-BAL will be recruited. When attending for FB-BAL, they will have a cough swab and an induced sputum sample obtained. The primary outcome will be the discordance of the pathogen yield from the cough swab and the induced sputum when compared with FB-BAL. Secondary outcomes will be the sensitivity of each sampling strategy, the success rate of the induced sputum in producing a usable sample and the tolerability of each of the three sampling strategies.

**Discussion:**

If either or both of the two less invasive airway sampling strategies are shown to be a useful alternative to FB-BAL, this will lead to more children with PBB having lower airway samples enabling targeted antibiotic prescribing. It would also reduce the need for FB, which is known to be burdensome for children and their families.

**Trial registration number:**

ISRCTN79883982.

**WHAT IS ALREADY KNOWN ON THIS TOPIC**

- Protracted bacterial bronchitis (PBB) is an endobronchial infection and the most common cause of chronic wet cough in children from the developed world.
- Identifying the causative organism in PBB allows a narrower spectrum antibiotic to be used which decreases the risk of antibiotic resistance.
- Children with PBB rarely expectorate sputum and the only widely used strategy to sample the lower airway is flexible bronchoscopy (FB). As this is invasive, it is reserved for severe or recurrent cases meaning most children with PBB are treated empirically with a broad-spectrum antibiotic.

**WHAT THIS STUDY ADDS**

- This study will investigate if a cough swab or an induced sputum sample is a useful alternative to bronchoalveolar lavage (BAL) samples obtained during flexible bronchoscopy (FB-BAL) for the identification of the causative organism(s) in children with PBB.

**HOW THIS STUDY MIGHT AFFECT RESEARCH, PRACTICE OR POLICY**

- If either or both of the two non-invasive strategies are shown to be a useful alternative to FB-BAL, this will lead to more children with PBB having lower airway samples enabling targeted antibiotic prescribing and reducing the need for FB.

## Introduction

Protracted bacterial bronchitis (PBB) is the leading cause of chronic wet cough in children from developed countries and the most common reason for referrals to UK Paediatric Respiratory clinics.^1 2^ It is caused by bacterial infection of the conducting airways.^3^ It is most prevalent in children aged 1–5 years but can occur up to the age of 10. The cough is persistent and troublesome. It affects the child’s sleep, school performance and physical activities.^4^ This has a detrimental effect on the child’s quality of life (QoL) and that of family members.^5^ Moreover, untreated PBB is associated with subsequent permanent airway damage (bronchiectasis)^6 7^ causing long-term morbidity and increasing healthcare utilisation.^8^

The original diagnostic criteria (now called PBB-micro) were: (1) wet cough >4 weeks, (2) culture of a respiratory pathogen from a lower airway sample and (3) cough cessation after course of appropriate oral antibiotic.^2^ Commonly causative pathogens include: *Haemophilus influenzae*, *Streptococcus pneumoniae*, *Moraxella catarrhalis* and *Staphylococcus aureus*.^9^ As children with PBB rarely expectorate sputum, the only widely used method to sample the lower airway is bronchoalveolar lavage obtained during flexible bronchoscopy (FB-BAL). This is invasive, requires a general anaesthetic and causes significant disruption to families.^10^ Due to the large number of children presenting to Paediatric Respiratory clinics with PBB, it is not practical to undertake FB-BAL in all cases. An alternative diagnostic criteria was therefore developed (PBB-clinical) in which the need for ‘culture of a respiratory pathogen from a lower airway sample’ was replaced with ‘absence of symptoms or signs of other causes of wet cough’.^11^ FB-BAL is now only undertaken in children with PBB if their symptoms fail to improve with the initial treatment or frequently relapse.^12^

Due to the issues in obtaining lower airway samples, the majority of children diagnosed with PBB are started on a treatment without microbiology data to inform the choice of antibiotic.^12^ This means a broad-spectrum antibiotic such as co-amoxiclav has to be used.^13^ When microbiology data are available, it allows a narrower spectrum antibiotic to be used which decreases the risk of antibiotic resistance.^14^ If a non-invasive, child-friendly method of sampling the lower airway was shown to be effective in providing microbiology data for children with PBB, it would mean less FB-BAL would be required reducing the disruption to families. It would also enable lower airway samples to be obtained in more children enabling antibiotic prescribing to be targeted, reducing antibiotic resistance. Data from trials in children with cystic fibrosis (CF)^15^ suggest that there may be a role for cough swabs or induced sputum samples in PBB, this cannot be assumed without undertaking a study.

### Objectives

The primary objective is the discordance in pathogen yield between cough swab/induced sputum and FB-BAL.

The secondary objectives are as follows:

1. To calculate the sensitivity of each sampling technique (cough swab, induced sputum and FB-BAL) to correctly identify all pathogens isolated from the lower airway in children with PBB.
2. To report the success rate of obtaining a usable induced sputum sample in children with PBB.
3. To report the tolerability of the three sampling measures (cough swab, induced sputum and FB-BAL).

### Outcomes

The primary outcome is the discordance in pathogen yield between cough swab/induced sputum and FB-BAL.

The secondary outcomes are as follows:

1. The sensitivity of each sampling technique estimated using triplicated (cough swab/induced sputum/FB-BAL) culture results as gold standard.
2. The success rate of obtaining a usable induced sputum sample in children with PBB as recorded by the physiotherapist in the study case report form (CRF).
3. The tolerability of the three sampling strategies (cough swab, induced sputum and FB-BAL) as recorded on Likert scale questionnaires.

### Trial design

CLASSIC PBB is a multicentre clinical trial in which children with PBB, who are referred for a clinically indicated FB-BAL, will also have a cough swab and an induced sputum sample obtained.

### Trial setting

This is a multicentre study taking place in four UK children’s hospitals: Alder Hey Children’s Hospital (Liverpool), Great North Children’s Hospital (Newcastle upon Tyne), Sheffield Children’s Hospital and Staffordshire Children’s Hospital at Royal Stoke (Stoke on Trent).

### Participants

A total of 131 children (aged 1–10 years) with a clinical diagnosis of PBB who have been referred for a clinically indicated FB-BAL will be recruited from the four centres in accordance with the criteria shown in table 1. Patients will be identified from the paediatric respiratory clinics by the delegated local study team and paediatric respiratory consultants. In the clinic, a member of the study team will discuss the study with the parent/guardian and provide the relevant participant information sheet(s) (PIS). After sufficient time for consideration, patients/parents/guardians who wish to participate will provide informed, written consent and assent when appropriate.

**Table 1:** Inclusion and exclusion criteria

Inclusion criteria	Exclusion criteria
Aged 1–10 years	Diagnosis of bronchiectasis, cystic fibrosis or immunodeficiency
Have a clinical diagnosis of PBB	FB-BAL being performed for a therapeutic indication (ie, lobar collapse) rather than for lower airway
Referred for a clinically indicated FB-BAL	Non-English speaker where translation facilities are insufficient to guarantee informed consent
Parent/guardian willing and able to give fully informed consent	Is taking part in another interventional study
Willing and able to comply with the study procedures	

BAL, bronchoalveolar lavage; FB, flexible bronchoscopy; PBB, protracted bacterial bronchitis.

### Trial assessments

The cough swab and induced sputum will be obtained on the same day and prior to the child’s FB-BAL by a delegated paediatric physiotherapist trained in the procedures. These procedures are aerosol generating so the local National Health Service (NHS) Trust guidelines regarding COVID-19 screening of children prior to admission and the use of personal protective equipment during the procedure will be followed.

#### Cough swab

The child will be instructed to tilt their head back and open their mouth wide. A sterile cotton swab will be placed at the back of the throat under the uvula and the child will be asked to cough. If the patient is too young to cough on command, the swab will be gently placed against the posterior pharynx to stimulate a cough. The sample will be labelled according to local guidelines and sent to the local microbiology.

#### Induced sputum

A 8 mL of 7% saline will be administered through a disposable oxygen-driven jet nebuliser set at a flow rate of 5 L/min for 15 min. Chest physiotherapy appropriate to the child’s age and ability to cooperate will be given during and after the nebulised therapy. Oropharyngeal suction will be used to obtain a sputum sample if the child is unable to spontaneously expectorate. The sample will be labelled according to local guidelines and sent to the local microbiology laboratory for culture. Oxygen saturations (SpO_2_) will be monitored before, during and after procedure. If the child develops wheeze or SpO_2_ drops<94%, inhaled or nebulised salbutamol will be delivered. The procedure will be discontinued if symptoms persist or SpO_2_ remains <94%.

#### FB-BAL

The FB-BAL is clinically indicated and will be performed as per standard of care and is therefore not a research procedure. It will be performed under general anaesthetic using the following methodology across all four sites. Once the bronchoscope is inserted, suction of secretions will be avoided before BAL to limit contamination with upper airway organisms. A single 10 mL aliquot lavage will be instilled and retrieved using a syringe attached to the bronchoscope channel in each of the six lobes (including lingula). The lavages will be undertaken in a specific order: right upper lobe, right middle lobe (RML), right lower lobe (RLL), left upper lobe, left lingular (LLi) and left lower lobe). The sample will be labelled according to local guidelines and sent to the local microbiology laboratory for culture.

#### Procedure tolerability

A four-question, Likert scale tolerability questionnaire will be completed after each of the three procedures. This will be done by the parent/guardian with input from the child when appropriate.

### Treatment of isolated organisms

The treatment of organisms identified from lower airway samples is not part of the study protocol. All results will be available for the local clinical team who will follow local guidance about the treatment.

### Microbiology samples

All microbiology samples (FB-BAL, cough swabs and induced sputum samples) will be handled and stored in-line with the UK Standards for Microbiology Investigations (UK SMI).

### Public and Patient involvement

CLASSIC PBB was developed to address the concerns of families related to the burden associated with FB-BAL. We undertook detailed interviews with the parents of 25 children with PBB who have undergone FB-BAL. They identified significant burden related to anxiety about the procedure, the need to take time off school/work and financial implications.^10^ These results highlighted the need to look for a non-invasive alternative to FB-BAL for children with PBB and have helped us decide on the topics for the parental assessment of tolerability. Three parent contributors helped develop the study methodology which was also presented to the Alder Hey Young Persons Advisory Group.

One of the parent contributors (EH) is PPIE lead for the study, a coapplicant on the funding application and coauthor on this paper. She has helped develop the PIS and consent/assent forms as well as being a member of the trial steering committee (TSC) where she will continue to be an advocate of parents and children. Once the study is complete, she will help in writing the plain English summary of the results.

### Statistical data analysis

#### Sample size calculation

The sample size was calculated using the McNemar’s test^16^ for paired proportions, informed by the comparison of pathogen yield from paired induced sputum and six-lobe FB-BAL samples in the CF-SPIT study.^15^ Assuming a proportion of discordant pairs of 0.37 we require a sample size of 110 to detect a ratio of discordant proportions (identification of an organism(s) on FB-BAL not isolated on induced sputum vs identification of organism(s) on induced sputum not identified on FB-BAL) of 3, with 90% power and 0.05 significance. If we assume the same induced sputum success rate as CF-SPIT (84%), we need a final sample size of 131. The sample size required to detect a difference between cough swab and FB-BAL is smaller due to a higher proportion of discordant pairs.

#### Primary outcome analysis

The organisms isolated from each participants cough swab will be compared with those isolated from their FB-BAL samples to identify concordance/discordance. The pathogen yield is concordant if the same organism(s) are identified in both samples or no organisms are identified in either. If the same organisms are not identified in both samples the pathogen yield is discordant. Discordance will be classified as CS^+^/FB-BAL^−^ when an organism is isolated on cough swab but not on FB-BAL or CS^−^/FB-BAL^+^ when an organism is isolated on FB-BAL but not on cough swab. This will be repeated with the FB-BAL organisms limited to those from a single lobe (RML) and two lobes (RML and LLi) to allow comparison of pathogen yield if a more limited FB-BAL sampling methodology had been used. The same analysis will be undertaken to identify concordance/discordance between the induced sputum and the FB-BAL samples.

We will use conditional logistic regression analyses to estimate the OR of discordant proportions for the paired cough swabs and FB-BAL samples (identification of an organism(s) on FB-BAL not isolated on cough swab vs identification of organism(s) on cough swab not identified on FB-BAL). This will be analysed separately using FB-BAL results from one, two and six lobes. A similar analysis will be undertaken for the paired induced sputum and FB-BAL samples.

#### Secondary outcomes analysis

1. We will estimate the sensitivity for each sampling technique against a combined gold standard consisting of all pathogens isolated from the triplicate samples (cough swab, sputum induction and six-lobe FB-BAL). A positive outcome will be defined as the ability of a single technique to identify all pathogens from the combined gold standard. This will enable us to quantify the ability of each technique to correctly detect all lower airway pathogens in any given patient. Two-sided 95% score CIs for sensitivity will be estimated.
2. We will use a logistic regression analysis to assess the effect of age on pathogen positivity and the success rate of sputum induction.
3. The Likert score for tolerability will be summarised using a frequency table stratified for age.

### Study oversight and monitoring

#### Trial management group

The trial management group will be responsible for the day-to-day management of the study. It will consist of the chief investigator (CI), principal investigators, statistician, clinical trial manager, data manager, data co-ordinator, qualityassurance manager and microbiology lead.

#### Trial Steering Committee

The TSC will provide supervision for the trial/study on behalf of the sponsor and funder. It will also provide expert advice independent of the CI and the Sponsor. The TSC will consist of an independent chair, two independent paediatric respiratory consultants, an independent statistician, the PPIE lead and the CI.

#### Study monitoring

Monitoring will be undertaken in accordance with the study monitoring plan. The monitor may review processes related to participant enrolment, eligibility, consent and adherence. They will also assess policies to protect participants, including reporting of harm and completeness, accuracy and timeliness of data collection. Monitoring may be carried out remotely by exploring the study dataset or performing site visits.

#### Data management

Data will be collected in accordance with the data management plan using CRFs. The completed CRFs will be returned to the data coordinator and inputted onto the REDCap database. Data quality checks will be conducted periodically to ensure accuracy of the primary and secondary outcomes.

#### Study progress

This 24 month trial opened on 01/10/2021. Recruitment started on 01/01/2022 and will be open for 17 months.

## Discussion

CLASSIC PBB will compare the pathogen yield from different lower airway sampling strategies in children with PBB. If either cough swab or induced sputum are shown to be useful alternatives to FB-BAL, it will enable more children with PBB to have the causative pathogen identified and therefore be prescribed targeted antibiotics. It would also reduce the need for FB-BAL in this condition which would be beneficial to children and their families due to the associated burden.

Cough swabs and induced sputum samples are not used routinely in children with PBB. They are, however, widely used in children with other respiratory illnesses who are unable to expectorate sputum. At UK paediatric CF) Centres, a cough swab is obtained at each out-patient appointment (2–3 monthly) as part of routine microbiological surveillance.^17^ They are easy to perform and well tolerated. The CF-SPIT study compared pathogen yield from cough swab, induced sputum and FB-BAL samples in children aged 6 months to 18 years with CF. They reported 69% of pathogens isolated at FB-BAL had been identified in cough swabs over the previous 12 months but a single cough swab was not representative of lower airway microbiology in CF. This supports the findings of a previous study which found the sensitivity of a single cough swabs to be 44% when compared with FB-BAL samples.^18^

Induced sputum has been shown to be a simple, cost effective, well tolerated and repeatable method of sampling the lower airway in children with CF.^15 19^ In the CF-SPIT study, the pathogen yield of 41 paired induced sputum and FB-BAL samples was compared.^15^ Of the 41 paired samples, 28 (68%) had a positive growth from at least one of the samples with 39 pathogens isolated in total. Induced sputum identified 27 (69%) pathogens, single-lobe FB-BAL identified 22 (56%), two-lobe FB-BAL 28 (72%) and six-lobe FB-BAL identified 33 (85%). Importantly, some pathogens were isolated from induced sputum and not FB-BAL reflecting the relative inability of FB-BAL to sample the larger airways. The authors of the CF-SPIT concluded that in children with CF, sputum induction is a credible surrogate for BAL and a substantial number of FB-BALs could be avoided if sputum induction is done first and pathogens appropriately treated.^15^

Although induced sputum sounds unpleasant, the success and tolerability rates in published cohorts is high. The three largest cohorts (CF-SPIT^15^ and two studies of children with suspected TB^20 21^ report 1099 attempts at induced sputum in 523 children aged 1 month to 16 years. A sputum sample was successfully obtained in 94% of procedures. Objective tolerance of the sputum induction procedure is good with CF-SPIT reporting no significant effects on respiratory rate, heart rate, or FEV_1_%. Subjective tolerance was also good. Likert scales rated tolerance high, with mean parent or patient scores of 8·55 (SD 1·65) and physiotherapist scores of 9·09 (1·76). During the 200 induced sputum reported in CF-SPIT, there was a low incidence of side-effects: 17 (9%) became upset, of which four (2%) could not complete the procedure; six (3%) had mild wheeze, of which two (1%) could not complete the procedure; three (2%) patients vomited during oropharyngeal suction and one (<1%) became transiently dizzy.

While induced sputum has been shown to be a useful alternative to FB-BAL in children with CF and TB it cannot be assumed this is the case for PBB. Children with PBB are younger than those with CF and the respiratory pathology is less suppurative. These factors potentially affect the likelihood of successfully obtaining samples.

## Supplementary Material

Reviewer comments

Author's manuscript

## Data Availability

No data are available.
