# Supplementary material for: Protocol for CLASSIC PBB: comparison of lower airway sampling strategies in children with protracted bacterial bronchitis
Source: BMJ Paediatr Open. 2022 Nov 21;6(1):e001722. doi: 10.1136/bmjpo-2022-001722 (PMC9680323; doi:10.1136/bmjpo-2022-001722)
Supplement: Author's manuscript [file bmjpo-2022-001722.draft_revisions.pdf]

BMJ Paediatrics Open

BMJ Paediatrics Open is committed to open peer review. As part of this commitment we make the peer review history of every article we publish publicly available.

When an article is published we post the peer reviewers' comments and the authors' responses online. We also post the versions of the paper that were used during peer review. These are the versions that the peer review comments apply to.

The versions of the paper that follow are the versions that were submitted during the peer review process. They are not the versions of record or the final published versions. They should not be cited or distributed as the published version of this manuscript.

BMJ Paediatrics Open is an open access journal and the full, final, typeset and author-corrected version of record of the manuscript is available on our site with no access controls, subscription charges or pay-per-view fees (http://bmjpaedsopen.bmj.com).

If you have any questions on BMJ Paediatrics Open's open peer review process please email info.bmjpo@bmj.com

BMJ Paediatrics Open

The protocol for CLASSIC PBB: Comparison of Lower Airway Sampling Strategies in Children with Protracted Bacterial Bronchitis

Journal:	*BMJ Paediatrics Open*
Manuscript ID	bmjpo-2022-001722
Article Type:	Original research
Date Submitted by the Author:	20-Oct-2022
Complete List of Authors:	Gilchrist, Francis; Keele University, Institute of Applied Clinical Science; University Hospitals of North Midlands NHS Trust, Paediatric Respiratory Medicine<br>Aspey, Mathew; University Hospitals of North Midlands NHS Trust, Research and Innovation Directorate<br>Bowler, Robert; University Hospitals of North Midlands NHS Trust, Research and Innovation Directorate<br>Brodlie, Malcolm; Newcastle University, Institute of Celular Medicine; Great North Children's Hospital, Paediatric Respiratory Medicine<br>Desai, Seema; University Hospitals of North Midlands NHS Trust, Department of Microbiology<br>Harris, Caroline; Great North Children's Hospital, Paediatric Respiratory Medicine<br>Hinton, Emily; University Hospitals of North Midlands NHS Trust, On Behalf of Patient and Public Involvement and Engagement for CLASSIC PBB<br>Kulkarni, Hemant; Sheffield Teaching Hospitals NHS Foundation Trust, Sheffield Children's Hosptial<br>Ogbolosingha, Aviva; University Hospitals of North Midlands NHS Trust, Research and Innovation Directorate<br>Sinha, Ian; Alder Hey Children's NHS Foundation Trust, Department of Respiratory Medicine<br>Solis-Trapala, Ivonne; Keele University, 10) School of Medicine<br>Stock, Joanne; University Hospitals of North Midlands NHS Trust, Paediatric Respiratory Medicine<br>Carroll, William; University Hospitals of North Midlands NHS Trust, Paediatric Respiratory Services; Keele University, Institute of Applied Clinical Services
Keywords:	Microbiology

SCHOLARONE™
Manuscripts

BMJ

I, the Submitting Author has the right to grant and does grant on behalf of all authors of the Work (as defined in the below author licence), an exclusive licence and/or a non-exclusive licence for contributions from authors who are: i) UK Crown employees; ii) where BMJ has agreed a CC-BY licence shall apply, and/or iii) in accordance with the terms applicable for US Federal Government officers or employees acting as part of their official duties; on a worldwide, perpetual, irrevocable, royalty-free basis to BMJ Publishing Group Ltd ("BMJ") its licensees and where the relevant Journal is co-owned by BMJ to the co-owners of the Journal, to publish the Work in this journal and any other BMJ products and to exploit all rights, as set out in our licence.

The Submitting Author accepts and understands that any supply made under these terms is made by BMJ to the Submitting Author unless you are acting as an employee on behalf of your employer or a postgraduate student of an affiliated institution which is paying any applicable article publishing charge ("APC") for Open Access articles. Where the Submitting Author wishes to make the Work available on an Open Access basis (and intends to pay the relevant APC), the terms of reuse of such Open Access shall be governed by a Creative Commons licence – details of these licences and which Creative Commons licence will apply to this Work are set out in our licence referred to above.

Other than as permitted in any relevant BMJ Author's Self Archiving Policies, I confirm this Work has not been accepted for publication elsewhere, is not being considered for publication elsewhere and does not duplicate material already published. I confirm all authors consent to publication of this Work and authorise the granting of this licence.

Confidential: For Review Only

The protocol for CLASSIC PBB: Comparison of Lower Airway Sampling Strategies in Children with Protracted Bacterial Bronchitis

Gilchrist FJ[1,2], Aspey M[3], Bowler R[3], Brodlie M[4,5], Desai S[6], Harris C[4], Hinton E[7], Kulkarni H[8], Ogbolosingha A[3], Sinha I[9], Solis-Trapala I[10], Stock J[1], Carroll WD[1,2]

1) Department of Paediatric Respiratory Medicine, Staffordshire Children's Hospital at Royal Stoke, University Hospitals of North Midlands NHS Trust, Stoke on Trent, ST4 6QG, UK
2) Institute of Applied Clinical Science, Keele University, Keele, ST5 5BG, UK
3) Research and Innovation Directorate, University Hospitals of North Midlands NHS Trust, Newcastle Road, Stoke-on-Trent, ST4 6QG, UK
4) Department of Paediatric Respiratory Medicine, Great North Children's Hospital, Newcastle upon Tyne Hospitals NHS Foundation Trust, Newcastle upon Tyne, NE1 4LP, UK
5) Translational and Clinical Research Institute, Faculty of Medical Sciences, Newcastle University, NE2 4HH, UK
6) Department of Microbiology, University Hospitals of North Midlands NHS Trust, Newcastle Road, Stoke-on-Trent, ST4 6QG, UK
7) On Behalf of Patient and Public Involvement and Engagement for CLASSIC PBB, Stoke on Trent, UK
8) Department of Paediatric Respiratory Medicine, Sheffield's Children's Hospital, Sheffield, S10 2TH, UK
9) Department of Paediatric Respiratory Medicine, Alder Hey Children's Hospital, Liverpool, L14 5AB, UK
10) School of Medicine, Keele University, Keele, ST5 5BG

What is already known on this topic?

- Protracted bacterial bronchitis is an endobronchial infection and the commonest cause of chronic wet cough in children from the developed world.
- Identifying the causative organism in PBB allows a narrower spectrum antibiotic to be used which decreases the risk of antibiotic resistance.
- Children with PBB rarely expectorate sputum and the only widely used strategy to sample the lower airway is flexible bronchoscopy. As this is invasive, it is reserved for severe or recurrent cases meaning most children with PBB are treated empirically with a broad-spectrum antibiotic.

What this study adds

- This study will investigate if a cough swab or an induced sputum sample is a useful alternative to bronchoalveolar lavage samples obtained during flexible bronchoscopy (FB-BAL) for the identification of the causative organism(s) in children with PBB.

How this study might affect research, practice or policy

- If either or both of the two non-invasive strategies are shown to be a useful alternative to FB-BAL, this will lead to more children with PBB having lower airway samples enabling targeted antibiotic prescribing and reducing the need for flexible bronchoscopy.

Confidential: For Review Only

Abstract

Background

Protracted Bacterial Bronchitis (PBB) is an endobronchial infection and a commonest cause of chronic wet cough in young children. It is treated with antibiotics which can only be targeted if the causative organism is known. As most affected children do not expectorate sputum, lower airway samples can only be obtained by bronchoalveolar lavage samples taken during flexible bronchoscopy (FB-BAL). This is invasive and is therefore reserved for children with severe or relapsing cases. Most children with PBB are treated empirically with broad spectrum antibiotics. CLASSIC PBB will compare the pathogen yield from two less invasive strategies with that from FB-BAL to see if they are comparable.

Methods

131 children with PBB from four UK centres referred FB-BAL will be recruited. When attending for FB-BAL, they will have a cough swab and an induced sputum sample obtained. The primary outcome will be the discordance of the pathogen yield from the cough swab and the induced sputum when compared to FB-BAL. Secondary outcomes will be the sensitivity of each sampling strategy, the success rate of the induced sputum in producing a usable sample and the tolerability of each of the three sampling strategies.

Discussion

If either or both of the two less invasive airway sampling strategies are shown to be a useful alternative to FB-BAL, this will lead to more children with PBB having lower airway samples enabling targeted antibiotic prescribing. It would also reduce the need for flexible bronchoscopy which is known to be burdensome for children and their families.

Trial Funding, REC Approval and Registration

CLASSIC PBB has been funded by an NIHR Research for Patient Benefit Grant (NIHR202272), approved by London Central Research Ethics Committee (IRAS Project ID: 229341) and registered on the ISRCTN Registry (ISRCTN79883982).

Keywords

Bronchoscopy, cough, microbiology, paediatrics, protracted bacterial bronchitis.

Confidential: For Review Only

Introduction

Protracted bacterial bronchitis (PBB) is the leading cause of chronic wet cough in children from developed countries and the commonest reason for referrals to UK Paediatric Respiratory clinics.[1,2] It is caused by bacterial infection of the conducting airways.[3] It is most prevalent in children aged 1-5 years but can occur up to the age of 10. The cough is persistent and troublesome. It affects the child's sleep, school performance and physical activities.[4] This has a detrimental effect on the child's quality of life (QoL) and that of family members.[5] Moreover, untreated PBB is associated with subsequent permanent airway damage (bronchiectasis)[6,7] causing long-term morbidity and increasing healthcare utilisation.[8]

The original diagnostic criteria (now called PBB-micro) were: (i) wet cough >4 weeks, (ii) culture of a respiratory pathogen from a lower airway sample and (iii) cough cessation after course of appropriate oral antibiotic.[2] Commonly causative pathogens include: *Haemophilus influenzae* (HiB), *Streptococcus pneumoniae*, *Moraxella catarrhalis* and *Staphylococcus aureus*.[9] As children with PBB rarely expectorate sputum, the only widely used method to sample the lower airway is bronchoalveolar lavage obtained during flexible bronchoscopy (FB-BAL). This is invasive, requires a general anaesthetic and causes significant disruption to families.[10] Due to the large number of children presenting to Paediatric Respiratory clinics with PBB, it is not practical to undertake FB-BAL in all cases. An alternative diagnostic criteria was therefore developed (PBB-clinical) in which the need for 'culture of a respiratory pathogen from a lower airway sample' was replaced with 'absence of symptoms or signs of other causes of wet cough'.[11] FB-BAL is now only undertaken in children with PBB if their symptoms fail to improve with the initial treatment or frequently relapse.[12]

Due to the issues in obtaining lower airway samples, the majority of children diagnosed with PBB are started on a treatment without microbiology data to inform the choice of antibiotic.[12] This means a broad spectrum antibiotic such as co-amoxiclav has to be used.[13] When microbiology data are available, it allows a narrower spectrum antibiotic to be used which decreases the risk of antibiotic resistance.[14] If a non-invasive, child-friendly method of sampling the lower airway was shown to be effective in providing microbiology data for children with PBB, it would mean less FB-BAL would be required reducing the disruption to families. It would also enable lower airway samples to be obtained in more children enabling antibiotic prescribing to be targeted, reducing antibiotic resistance. Data from trials in children with cystic fibrosis (CF)[15] suggest there may be a role for cough swabs or induced sputum samples in PBB, this cannot be assumed without undertaking a study.

Objectives

The primary objective is the discordance in pathogen yield between cough swab / induced sputum and FB-BAL.

The secondary objectives are:

1. To calculate the sensitivity of each sampling technique (cough swab, induced sputum and FB-BAL) to correctly identify all pathogens isolated from the lower airway in children with PBB
2. To report the success rate of obtaining a usable induced sputum sample in children with PBB
3. To report the tolerability of the three sampling measures (cough swab, induced sputum and FB-BAL)

https://mc.manuscriptcentral.com/bmjpo

Outcomes

The primary outcome is the discordance in pathogen yield between cough swab / induced sputum and FB-BAL.

The secondary outcomes are:

1. The sensitivity of each sampling technique estimated using triplicated (cough swab / induced sputum / FB-BAL) culture results as gold-standard.
2. The success rate of obtaining a usable induced sputum sample in children with PBB as recorded by the physiotherapist in the study case report form (CRF)
3. The tolerability of the three sampling strategies (cough swab, induced sputum and FB-BAL) as recorded on Likert scale questionnaires

Trial Design

CLASSIC PBB is a multi-centre clinical trial in which children with PBB, who are referred for a clinically indicated FB-BAL, will also have a cough swab and an induced sputum sample obtained.

Trial Setting

This is a multi-centre study taking place in four UK children's hospitals: Alder Hey Children's Hospital (Liverpool), Great North Children's Hospital (Newcastle upon Tyne), Sheffield Children's Hospital and Staffordshire Children's Hospital at Royal Stoke (Stoke on Trent).

Participants

A total of 131 children (aged 1-10 years) with a clinical diagnosis of PBB who have been referred for a clinically indicated FB-BAL will be recruited from the four centres in accordance with the criteria shown in Table 1. Patients will be identified from the Paediatric Respiratory Clinics by the delegated local study team and Paediatric Respiratory Consultants. In the clinic, a member of the study team will discuss the study with the parent / guardian and provide the relevant Participant Information Sheet(s) (PIS). After sufficient time for consideration, patients/parents/guardians who wish to participate will provide informed, written consent and assent when appropriate.

Table 1: Inclusion and exclusion criteria

Inclusion Criteria	Exclusion Criteria
Aged 1-10 years	Diagnosis of bronchiectasis, cystic fibrosis or immunodeficiency
Have a clinical diagnosis of PBB	FB-BAL being performed for a therapeutic indication (i.e. lobar collapse) rather than for lower airway
Referred for a clinically indicated FB-BAL	Non-English speaker where translation facilities are insufficient to guarantee informed consent
Parent/Guardian willing and able to give fully informed consent	Is taking part in another interventional study
Willing and able to comply with the study procedures	

Confidential: For Review Only

Trial Assessments

The cough swab and induced sputum will be obtained on the same day and prior to the child's FB-BAL by a delegated paediatric physiotherapist trained in the procedures. These procedures are aerosol generating so the local NHS Trust guidelines regarding COVID-19 screening of children prior to admission and the use of personal protective equipment during the procedure will be followed.

Cough Swab

The child will be instructed to tilt their head back and open their mouth wide. A sterile cotton swab will be placed at the back of the throat under the uvula and the child will be asked to cough. If the patient is too young to cough on command, the swab will be gently placed against the posterior pharynx to stimulate a cough. The sample will be labelled according to local guidelines and sent to the local microbiology.

Induced Sputum

8ml of 7% saline will be administered through a disposable oxygen-driven jet nebuliser set at a flow rate of 5L/minute for 15 minutes. Chest physiotherapy appropriate to the child's age and ability to cooperate will be given during and after the nebulised therapy. Oropharyngeal suction will be used to obtain a sputum sample if the child is unable to spontaneously expectorate. The sample will be labelled according to local guidelines and sent to the local microbiology laboratory for culture. Oxygen saturations (SpO_2) will be monitored before, during and after procedure. If the child develops wheeze or SpO_2 drops <94%, inhaled or nebulised salbutamol will be delivered. The procedure will be discontinued if symptoms persist or SpO_2 remains <94%.

FB-BAL

The FB-BAL is clinically indicated and will be performed as per standard of care and is therefore not a research procedure. It will be performed under general anaesthetic using the following methodology across all four sites. Once the bronchoscope is inserted, suction of secretions will be avoided before BAL to limit contamination with upper airway organisms. A single 10ml aliquot lavage will be instilled and retrieved using a syringe attached to the bronchoscope channel in each of the six lobes (including lingula). The lavages will be undertaken in a specific order: right upper lobe (RUL), right middle lobe (RML), right lower lobe (RLL), left upper lobe (LUL), left lingular (LLi) and left lower lobe (LLL). The sample will be labelled according to local guidelines and sent to the local microbiology laboratory for culture.

Procedure Tolerability

A four question, Likert scale tolerability questionnaire will be completed after each of the three procedures. This will be done by the parent / guardian with input from the child when appropriate.

Treatment of isolated organisms

The treatment of organisms identified from lower airway samples is not part of the study protocol. All results will be available for the local clinical team who will follow local guidance about the treatment.

Confidential: For Review Only

Microbiology Samples

All microbiology samples (FB-BAL, cough swabs and induced sputum samples) will be handled and stored in-line with the UK Standards for Microbiology Investigations (UK SMI).

Public and Patient Involvement

CLASSIC PBB was developed to address the concerns of families related to the burden associated with FB-BAL. We undertook detailed interviews with the parents of 25 children with PBB who have undergone FB-BAL. They identified significant burden related to anxiety about the procedure, the need to take time off school / work and financial implications.[10] These results highlighted the need to look for a non-invasive alternative to FB-BAL for children with PBB and have helped us decide on the topics for the parental assessment of tolerability. Three parent contributors helped develop the study methodology which was also presented to the Alder Hey Young Persons Advisory Group (YPAG).

One of the parent contributors (EH) is PPIE lead for the study, a co-applicant on the funding application and co-author on this paper. She has helped develop the PIS and consent / assent forms as well as being a member of the Trial Steering Committee where she will continue to be an advocate of parents and children. Once the study is complete, she will help in writing the plain English summary of the results.

Statistical Data Analysis

Sample Size Calculation

The sample size was calculated using the McNemar's test[16] for paired proportions, informed by the comparison of pathogen yield from paired induced sputum and six-lobe FB-BAL samples in the CF-SPIT study.[15] Assuming a proportion of discordant pairs of 0.37 we require a sample size of 110 to detect a ratio of discordant proportions (identification of an organism[s] on FB-BAL not isolated on induced sputum vs identification of organism[s] on induced sputum not identified on FB-BAL) of 3, with 90% power and 0.05 significance. If we assume the same induced sputum success rate as CF-SPIT (84%) we need a final sample size of 131. The sample size required to detect a difference between cough swab and FB-BAL is smaller due to a higher proportion of discordant pairs.

Primary Outcome Analysis

The organisms isolated from each participants cough swab will be compared to those isolated from their FB-BAL samples to identify concordance / discordance. The pathogen yield is concordant if the same organism(s) are identified in both samples or no organisms are identified in either. If the same organisms are not identified in both samples the pathogen yield is discordant. Discordance will be classified as CS^+/FB-BAL^- when an organism is isolated on cough swab but not on FB-BAL or CS^-/FB-BAL^+ when an organism is isolated on FB-BAL but not on cough swab. This will be repeated with the FB-BAL organisms limited to those from a single lobe (RML) and two lobes (RML & LLi) to allow comparison of pathogen yield if a more limited FB-BAL sampling methodology had been used. The same analysis will be undertaken to identify concordance / discordance between the induced sputum and the FB-BAL samples.

Confidential: For Review Only

We will use conditional logistic regression analyses to estimate the odds ratio of discordant proportions for the paired cough swabs and FB-BAL samples (identification of an organism[s] on FB-BAL not isolated on cough swab versus identification of organism[s] on cough swab not identified on FB-BAL). This will be analysed separately using FB-BAL results from one, two and six lobes. A similar analysis will be undertaken for the paired induced sputum and FB-BAL samples.

Secondary Outcomes Analysis

1. We will estimate the sensitivity for each sampling technique against a combined gold standard consisting of all pathogens isolated from the triplicate samples (cough swab, sputum induction and six-lobe FB-BAL). A positive outcome will be defined as the ability of a single technique to identify all pathogens from the combined gold standard. This will enable us to quantify the ability of each technique to correctly detect all lower airway pathogens in any given patient. Two-sided 95% score confidence intervals for sensitivity will be estimated.
2. We will use a logistic regression analysis to assess the effect of age on pathogen positivity and the success rate of sputum induction.
3. The Likert score for tolerability will be summarised using a frequency table stratified for age.

Study Oversight and Monitoring

Trial Management Group (TMG)

The TMG will be responsible for the day-to-day management of the study. It will consist of the Chief Investigator (CI), Principal Investigators (PIs), Statistician, Clinical Trial Manager, Data Manager, Data Co-ordinator, Quality Assurance Manager and Microbiology Lead.

Trial Steering Committee (TSC)

The TSC will provide supervision for the trial / study on behalf of the Sponsor and Funder. It will also provide expert advice independent of the CI and the Sponsor. The TSC will consist of an independent chair, two independent paediatric respiratory consultants, an independent statistician, the PPIE Lead and the CI.

Study Monitoring

Monitoring will be undertaken in accordance with the Study Monitoring Plan. The monitor may review processes related to participant enrolment, eligibility, consent and adherence. They will also assess policies to protect participants, including reporting of harm and completeness, accuracy, and timeliness of data collection. Monitoring may be carried out remotely by exploring the study dataset or performing site visits.

Data Management

Data will be collected in accordance with the Data Management Plan using CRFs. The completed CRFs will be returned to the Data Coordinator and inputted onto the REDCap database. Data quality checks will be conducted periodically to ensure accuracy of the primary and secondary outcomes.

Confidential: For Review Only

Funding, Ethical Approval and Trial Registration

Funding

CLASSIC PBB has been funded by an NIHR Research for Patient Benefit Grant (NIHR202272)

REC Approval

CLASSIC PBB was approved by London Central Research Ethics Committee (IRAS Project ID: 229341).

Clinical Trial Registration

CLASSIC PBB has been registered on the ISRCTN Registry (ISRCTN79883982)

Study Progress

This 24-month trial opened on 01/10/2021. Recruitment started on 01/01/2022 and will be open for 17 months.

Discussion

CLASSIC PBB is the first study to compare the pathogen yield from different lower airway sampling strategies in children with PBB. If either cough swab or induced sputum are shown to be useful alternatives to FB-BAL, it will enable more children with PBB to have the causative pathogen identified and therefore be prescribed targeted antibiotics. It would also reduce the need for FB-BAL in this condition which would be beneficial to children and their families due to the associated burden.

Cough swabs and induced sputum samples are not used routinely in children with PBB. They are, however, widely used in children with other respiratory illnesses who are unable to expectorate sputum. At UK paediatric cystic fibrosis (CF) Centres, a cough swab is obtained at each out-patient appointment (2-3 monthly) as part of routine microbiological surveillance.[17] They are easy to perform and well tolerated. The CF-SPIT study compared pathogen yield from cough swab, induced sputum and FB-BAL samples in children aged 6 months to 18 years with CF. They reported 69% of pathogens isolated at FB-BAL had been identified in cough swabs over the previous 12 months but a single cough swab was not representative of lower airway microbiology in CF. This supports the findings of a previous study which found the sensitivity of a single cough swabs to be 44% when compared to FB-BAL samples.[18]

Induced sputum has been shown to be a simple, cost effective, well tolerated and repeatable method of sampling the lower airway in children with CF.[15,19] In the CF-SPIT study, the pathogen yield of 41 paired induced sputum and FB-BAL samples was compared.[15] Of the 41 paired samples, 28 (68%) had a positive growth from at least one of the samples with 39 pathogens isolated in total. Induced sputum identified 27 (69%) pathogens, single-lobe FB-BAL identified 22 (56%), two-lobe FB-BAL 28 (72%) and six-lobe FB-BAL identified 33 (85%). Importantly, some pathogens were isolated from induced sputum and not FB-BAL reflecting the relative inability of FB-BAL to sample the larger airways. The authors of the CF-SPIT concluded that in children with CF, sputum induction is a credible surrogate for bronchoalveolar lavage and a substantial number of FB-BALs could be avoided if sputum induction is done first and pathogens appropriately treated.[15]

Confidential: For Review Only

Although induced sputum sounds unpleasant, the success and tolerability rates in published cohorts is high. The three largest cohorts (CF-SPIT[15] and two studies of children with suspected TB[20,21]) report 1,099 attempts at induced sputum in 523 children aged 1 month to 16 years. A sputum sample was successfully obtained in 94% of procedures. Objective tolerance of the sputum induction procedure is good with CF-SPIT reporting no significant effects on respiratory rate, heart rate, or FEV_1%. Subjective tolerance was also good. Likert scales rated tolerance high, with mean parent or patient scores of 8·55 (SD 1·65) and physiotherapist scores of 9·09 (1·76). During the 200 induced sputum reported in CF-SPIT, there was a low incidence of side-effects: 17 (9%) became upset, of which four (2%) could not complete the procedure; six (3%) had mild wheeze, of which two (1%) could not complete the procedure; three (2%) patients vomited during oropharyngeal suction and one (<1%) became transiently dizzy.

Whilst induced sputum has been shown to be a useful alternative to FB-BAL in children with CF and TB it cannot be assumed this is the case for PBB. Children with PBB are younger than those with CF and the respiratory pathology is less suppurative. These factors potentially affect the likelihood of successfully obtaining samples.

Confidential: For Review Only

References

1. Brodlie M, Graham C, McKean MC. Childhood cough. BMJ 2012;344:e1177.

2. Marchant JM, Masters IB, Taylor SM, Cox NC, Seymour GJ, Chang AB. Evaluation and outcome of young children with chronic cough. Chest 2006;129(5):1132–41.

3. Craven V, Everard ML. Protracted bacterial bronchitis: reinventing an old disease. Arch Dis Child 2013;98(1):72–6.

4. Chang AB, Robertson CF, Van Asperen PP, et al. A multicenter study on chronic cough in children : burden and etiologies based on a standardized management pathway. Chest 2012;142(4):943–50.

5. Newcombe PA, Sheffield JK, Chang AB. Parent cough-specific quality of life: development and validation of a short form. J Allergy Clin Immunol 2013;131(4):1069–74.

6. Chang AB, Redding GJ, Everard ML. Chronic wet cough: Protracted bronchitis, chronic suppurative lung disease and bronchiectasis. Pediatr Pulmonol 2008;43(6):519–31.

7. Wurzel DF, Marchant JM, Yerkovich ST, et al. Protracted Bacterial Bronchitis in Children: Natural History and Risk Factors for Bronchiectasis. Chest 2016;150(5):1101–8.

8. Chang AB, Bush A, Grimwood K. Bronchiectasis in children: diagnosis and treatment. Lancet 2018;392(10150):866–79.

9. Narang R, Bakewell K, Peach J, et al. Bacterial distribution in the lungs of children with protracted bacterial bronchitis. PLoS ONE 2014;9(9):e108523.

10. Swift JA, Carroll WD, Gilchrist FJ. Single-centre survey of parents regarding the hidden burden of paediatric flexible bronchoscopy. BMJ Paediatr Open 2021;5(1):e000991.

11. Chang AB, Upham JW, Masters IB, et al. Protracted bacterial bronchitis: The last decade and the road ahead. Pediatr Pulmonol 2016;51(3):225–42.

12. Gilchrist FJ. An approach to the child with a wet cough. Paediatr Respir Rev 2019;31:75–81.

13. Marchant J, Masters IB, Champion A, Petsky H, Chang AB. Randomised controlled trial of amoxycillin clavulanate in children with chronic wet cough. Thorax 2012;67(8):689–93.

14. Pritchard MG, Lenney W, Gilchrist FJ. Outcomes in children with protracted bacterial bronchitis confirmed by bronchoscopy. Arch Dis Child 2015;100(1):112.

15. Ronchetti K, Tame J-D, Paisey C, et al. The CF-Sputum Induction Trial (CF-SpIT) to assess lower airway bacterial sampling in young children with cystic fibrosis: a prospective internally controlled interventional trial. Lancet Respir Med 2018;6(6):461–71.

16. Julious SA, Campbell MJ, Altman DG. Estimating sample sizes for continuous, binary, and ordinal outcomes in paired comparisons: practical hints. J Biopharm Stat 1999;9(2):241–51.

17. Forton JT. Detecting respiratory infection in children with cystic fibrosis: Cough swab, sputum induction or bronchoalveolar lavage. Paediatr Respir Rev 2019;31:28–31.

Confidential: For Review Only

18. Rosenfeld M, Emerson J, Accurso F, et al. Diagnostic accuracy of oropharyngeal cultures in infants and young children with cystic fibrosis. Pediatr Pulmonol 1999;28(5):321–8.

19. Al-Saleh S, Dell SD, Grasemann H, et al. Sputum induction in routine clinical care of children with cystic fibrosis. J Pediatr 2010;157(6):1006-1011.e1.

20. Zar HJ, Tannenbaum E, Apolles P, Roux P, Hanslo D, Hussey G. Sputum induction for the diagnosis of pulmonary tuberculosis in infants and young children in an urban setting in South Africa. Arch Dis Child 2000;82(4):305–8.

21. Zar HJ, Hanslo D, Apolles P, Swingler G, Hussey G. Induced sputum versus gastric lavage for microbiological confirmation of pulmonary tuberculosis in infants and young children: a prospective study. Lancet 2005;365(9454):130–4.

Confidential: For Review Only

BMJ Paediatrics Open

The protocol for CLASSIC PBB: Comparison of Lower Airway Sampling Strategies in Children with Protracted Bacterial Bronchitis

Journal:	*BMJ Paediatrics Open*
Manuscript ID	bmjpo-2022-001722.R1
Article Type:	Protocol
Date Submitted by the Author:	26-Oct-2022

Complete List of Authors:	Gilchrist, Francis; Keele University, Institute of Applied Clinical Science; University Hospitals of North Midlands NHS Trust, Paediatric Respiratory Medicine<br>Aspey, Mathew; University Hospitals of North Midlands NHS Trust, Research and Innovation Directorate<br>Bowler, Robert; University Hospitals of North Midlands NHS Trust, Research and Innovation Directorate<br>Brodlie, Malcolm; Newcastle University, Institute of Celular Medicine; Great North Children's Hospital, Paediatric Respiratory Medicine<br>Desai, Seema; University Hospitals of North Midlands NHS Trust, Department of Microbiology<br>Harris, Caroline; Great North Children's Hospital, Paediatric Respiratory Medicine<br>Hinton, Emily; University Hospitals of North Midlands NHS Trust, On Behalf of Patient and Public Involvement and Engagement for CLASSIC PBB<br>Kulkarni, Hemant; Sheffield Teaching Hospitals NHS Foundation Trust, Sheffield Children's Hosptial<br>Ogbolosingha, Aviva; University Hospitals of North Midlands NHS Trust, Research and Innovation Directorate<br>Sinha, Ian; Alder Hey Children's NHS Foundation Trust, Department of Respiratory Medicine<br>Solis-Trapala, Ivonne; Keele University, 10) School of Medicine<br>Stock, Joanne; University Hospitals of North Midlands NHS Trust, Paediatric Respiratory Medicine<br>Carroll, William; University Hospitals of North Midlands NHS Trust, Paediatric Respiratory Services; Keele University, Institute of Applied Clinical Services

Keywords:	Microbiology

SCHOLARONE™ Manuscripts

I, the Submitting Author has the right to grant and does grant on behalf of all authors of the Work (as defined in the below author licence), an exclusive licence and/or a non-exclusive licence for contributions from authors who are: i) UK Crown employees; ii) where BMJ has agreed a CC-BY licence shall apply, and/or iii) in accordance with the terms applicable for US Federal Government officers or employees acting as part of their official duties; on a worldwide, perpetual, irrevocable, royalty-free basis to BMJ Publishing Group Ltd ("BMJ") its licensees and where the relevant Journal is co-owned by BMJ to the co-owners of the Journal, to publish the Work in this journal and any other BMJ products and to exploit all rights, as set out in our licence.

The Submitting Author accepts and understands that any supply made under these terms is made by BMJ to the Submitting Author unless you are acting as an employee on behalf of your employer or a postgraduate student of an affiliated institution which is paying any applicable article publishing charge ("APC") for Open Access articles. Where the Submitting Author wishes to make the Work available on an Open Access basis (and intends to pay the relevant APC), the terms of reuse of such Open Access shall be governed by a Creative Commons licence – details of these licences and which Creative Commons licence will apply to this Work are set out in our licence referred to above.

Other than as permitted in any relevant BMJ Author's Self Archiving Policies, I confirm this Work has not been accepted for publication elsewhere, is not being considered for publication elsewhere and does not duplicate material already published. I confirm all authors consent to publication of this Work and authorise the granting of this licence.

Confidential: For Review Only

The protocol for CLASSIC PBB: Comparison of Lower Airway Sampling Strategies in Children with Protracted Bacterial Bronchitis

Gilchrist FJ[1,2], Aspey M[3], Bowler R[3], Brodlie M[4,5], Desai S[6], Harris C[4], Hinton E[7], Kulkarni H[8], Ogbolosingha A[3], Sinha I[9], Solis-Trapala I[10], Stock J[1], Carroll WD[1,2]

1) Department of Paediatric Respiratory Medicine, Staffordshire Children's Hospital at Royal Stoke, University Hospitals of North Midlands NHS Trust, Stoke on Trent, ST4 6QG, UK
2) Institute of Applied Clinical Science, Keele University, Keele, ST5 5BG, UK
3) Research and Innovation Directorate, University Hospitals of North Midlands NHS Trust, Newcastle Road, Stoke-on-Trent, ST4 6QG, UK
4) Department of Paediatric Respiratory Medicine, Great North Children's Hospital, Newcastle upon Tyne Hospitals NHS Foundation Trust, Newcastle upon Tyne, NE1 4LP, UK
5) Translational and Clinical Research Institute, Faculty of Medical Sciences, Newcastle University, NE2 4HH, UK
6) Department of Microbiology, University Hospitals of North Midlands NHS Trust, Newcastle Road, Stoke-on-Trent, ST4 6QG, UK
7) On Behalf of Patient and Public Involvement and Engagement for CLASSIC PBB, Stoke on Trent, UK
8) Department of Paediatric Respiratory Medicine, Sheffield's Children's Hospital, Sheffield, S10 2TH, UK
9) Department of Paediatric Respiratory Medicine, Alder Hey Children's Hospital, Liverpool, L14 5AB, UK
10) School of Medicine, Keele University, Keele, ST5 5BG

What is already known on this topic?

- Protracted bacterial bronchitis is an endobronchial infection and the commonest cause of chronic wet cough in children from the developed world.
- Identifying the causative organism in PBB allows a narrower spectrum antibiotic to be used which decreases the risk of antibiotic resistance.
- Children with PBB rarely expectorate sputum and the only widely used strategy to sample the lower airway is flexible bronchoscopy. As this is invasive, it is reserved for severe or recurrent cases meaning most children with PBB are treated empirically with a broad-spectrum antibiotic.

What this study adds

- This study will investigate if a cough swab or an induced sputum sample is a useful alternative to bronchoalveolar lavage samples obtained during flexible bronchoscopy (FB-BAL) for the identification of the causative organism(s) in children with PBB.

How this study might affect research, practice or policy

- If either or both of the two non-invasive strategies are shown to be a useful alternative to FB-BAL, this will lead to more children with PBB having lower airway samples enabling targeted antibiotic prescribing and reducing the need for flexible bronchoscopy.

Confidential: For Review Only

Abstract

Background

Protracted Bacterial Bronchitis (PBB) is an endobronchial infection and a commonest cause of chronic wet cough in young children. It is treated with antibiotics which can only be targeted if the causative organism is known. As most affected children do not expectorate sputum, lower airway samples can only be obtained by bronchoalveolar lavage samples taken during flexible bronchoscopy (FB-BAL). This is invasive and is therefore reserved for children with severe or relapsing cases. Most children with PBB are treated empirically with broad spectrum antibiotics. CLASSIC PBB will compare the pathogen yield from two less invasive strategies with that from FB-BAL to see if they are comparable.

Methods

131 children with PBB from four UK centres referred FB-BAL will be recruited. When attending for FB-BAL, they will have a cough swab and an induced sputum sample obtained. The primary outcome will be the discordance of the pathogen yield from the cough swab and the induced sputum when compared to FB-BAL. Secondary outcomes will be the sensitivity of each sampling strategy, the success rate of the induced sputum in producing a usable sample and the tolerability of each of the three sampling strategies.

Discussion

If either or both of the two less invasive airway sampling strategies are shown to be a useful alternative to FB-BAL, this will lead to more children with PBB having lower airway samples enabling targeted antibiotic prescribing. It would also reduce the need for flexible bronchoscopy which is known to be burdensome for children and their families.

Trial Funding, REC Approval and Registration

CLASSIC PBB has been funded by an NIHR Research for Patient Benefit Grant (NIHR202272), approved by London Central Research Ethics Committee (IRAS Project ID: 229341) and registered on the ISRCTN Registry (ISRCTN79883982).

Keywords

Bronchoscopy, cough, microbiology, paediatrics, protracted bacterial bronchitis.

Confidential: For Review Only

Introduction

Protracted bacterial bronchitis (PBB) is the leading cause of chronic wet cough in children from developed countries and the commonest reason for referrals to UK Paediatric Respiratory clinics.[1,2] It is caused by bacterial infection of the conducting airways.[3] It is most prevalent in children aged 1-5 years but can occur up to the age of 10. The cough is persistent and troublesome. It affects the child's sleep, school performance and physical activities.[4] This has a detrimental effect on the child's quality of life (QoL) and that of family members.[5] Moreover, untreated PBB is associated with subsequent permanent airway damage (bronchiectasis)[6,7] causing long-term morbidity and increasing healthcare utilisation.[8]

The original diagnostic criteria (now called PBB-micro) were: (i) wet cough >4 weeks, (ii) culture of a respiratory pathogen from a lower airway sample and (iii) cough cessation after course of appropriate oral antibiotic.[2] Commonly causative pathogens include: *Haemophilus influenzae* (HiB), *Streptococcus pneumoniae*, *Moraxella catarrhalis* and *Staphylococcus aureus*.[9] As children with PBB rarely expectorate sputum, the only widely used method to sample the lower airway is bronchoalveolar lavage obtained during flexible bronchoscopy (FB-BAL). This is invasive, requires a general anaesthetic and causes significant disruption to families.[10] Due to the large number of children presenting to Paediatric Respiratory clinics with PBB, it is not practical to undertake FB-BAL in all cases. An alternative diagnostic criteria was therefore developed (PBB-clinical) in which the need for 'culture of a respiratory pathogen from a lower airway sample' was replaced with 'absence of symptoms or signs of other causes of wet cough'.[11] FB-BAL is now only undertaken in children with PBB if their symptoms fail to improve with the initial treatment or frequently relapse.[12]

Due to the issues in obtaining lower airway samples, the majority of children diagnosed with PBB are started on a treatment without microbiology data to inform the choice of antibiotic.[12] This means a broad spectrum antibiotic such as co-amoxiclav has to be used.[13] When microbiology data are available, it allows a narrower spectrum antibiotic to be used which decreases the risk of antibiotic resistance.[14] If a non-invasive, child-friendly method of sampling the lower airway was shown to be effective in providing microbiology data for children with PBB, it would mean less FB-BAL would be required reducing the disruption to families. It would also enable lower airway samples to be obtained in more children enabling antibiotic prescribing to be targeted, reducing antibiotic resistance. Data from trials in children with cystic fibrosis (CF)[15] suggest there may be a role for cough swabs or induced sputum samples in PBB, this cannot be assumed without undertaking a study.

Objectives

The primary objective is the discordance in pathogen yield between cough swab / induced sputum and FB-BAL.

The secondary objectives are:

1. To calculate the sensitivity of each sampling technique (cough swab, induced sputum and FB-BAL) to correctly identify all pathogens isolated from the lower airway in children with PBB
2. To report the success rate of obtaining a usable induced sputum sample in children with PBB
3. To report the tolerability of the three sampling measures (cough swab, induced sputum and FB-BAL)

https://mc.manuscriptcentral.com/bmjpo

Outcomes

The primary outcome is the discordance in pathogen yield between cough swab / induced sputum and FB-BAL.

The secondary outcomes are:

1. The sensitivity of each sampling technique estimated using triplicated (cough swab / induced sputum / FB-BAL) culture results as gold-standard.
2. The success rate of obtaining a usable induced sputum sample in children with PBB as recorded by the physiotherapist in the study case report form (CRF)
3. The tolerability of the three sampling strategies (cough swab, induced sputum and FB-BAL) as recorded on Likert scale questionnaires

Trial Design

CLASSIC PBB is a multi-centre clinical trial in which children with PBB, who are referred for a clinically indicated FB-BAL, will also have a cough swab and an induced sputum sample obtained.

Trial Setting

This is a multi-centre study taking place in four UK children's hospitals: Alder Hey Children's Hospital (Liverpool), Great North Children's Hospital (Newcastle upon Tyne), Sheffield Children's Hospital and Staffordshire Children's Hospital at Royal Stoke (Stoke on Trent).

Participants

A total of 131 children (aged 1-10 years) with a clinical diagnosis of PBB who have been referred for a clinically indicated FB-BAL will be recruited from the four centres in accordance with the criteria shown in Table 1. Patients will be identified from the Paediatric Respiratory Clinics by the delegated local study team and Paediatric Respiratory Consultants. In the clinic, a member of the study team will discuss the study with the parent / guardian and provide the relevant Participant Information Sheet(s) (PIS). After sufficient time for consideration, patients/parents/guardians who wish to participate will provide informed, written consent and assent when appropriate.

Table 1: Inclusion and exclusion criteria

Inclusion Criteria	Exclusion Criteria
Aged 1-10 years	Diagnosis of bronchiectasis, cystic fibrosis or immunodeficiency
Have a clinical diagnosis of PBB	FB-BAL being performed for a therapeutic indication (i.e. lobar collapse) rather than for lower airway
Referred for a clinically indicated FB-BAL	Non-English speaker where translation facilities are insufficient to guarantee informed consent
Parent/Guardian willing and able to give fully informed consent	Is taking part in another interventional study
Willing and able to comply with the study procedures	

Confidential: For Review Only

Trial Assessments

The cough swab and induced sputum will be obtained on the same day and prior to the child's FB-BAL by a delegated paediatric physiotherapist trained in the procedures. These procedures are aerosol generating so the local NHS Trust guidelines regarding COVID-19 screening of children prior to admission and the use of personal protective equipment during the procedure will be followed.

Cough Swab

The child will be instructed to tilt their head back and open their mouth wide. A sterile cotton swab will be placed at the back of the throat under the uvula and the child will be asked to cough. If the patient is too young to cough on command, the swab will be gently placed against the posterior pharynx to stimulate a cough. The sample will be labelled according to local guidelines and sent to the local microbiology.

Induced Sputum

8ml of 7% saline will be administered through a disposable oxygen-driven jet nebuliser set at a flow rate of 5L/minute for 15 minutes. Chest physiotherapy appropriate to the child's age and ability to cooperate will be given during and after the nebulised therapy. Oropharyngeal suction will be used to obtain a sputum sample if the child is unable to spontaneously expectorate. The sample will be labelled according to local guidelines and sent to the local microbiology laboratory for culture. Oxygen saturations (SpO_2) will be monitored before, during and after procedure. If the child develops wheeze or SpO_2 drops <94%, inhaled or nebulised salbutamol will be delivered. The procedure will be discontinued if symptoms persist or SpO_2 remains <94%.

FB-BAL

The FB-BAL is clinically indicated and will be performed as per standard of care and is therefore not a research procedure. It will be performed under general anaesthetic using the following methodology across all four sites. Once the bronchoscope is inserted, suction of secretions will be avoided before BAL to limit contamination with upper airway organisms. A single 10ml aliquot lavage will be instilled and retrieved using a syringe attached to the bronchoscope channel in each of the six lobes (including lingula). The lavages will be undertaken in a specific order: right upper lobe (RUL), right middle lobe (RML), right lower lobe (RLL), left upper lobe (LUL), left lingular (LLi) and left lower lobe (LLL). The sample will be labelled according to local guidelines and sent to the local microbiology laboratory for culture.

Procedure Tolerability

A four question, Likert scale tolerability questionnaire will be completed after each of the three procedures. This will be done by the parent / guardian with input from the child when appropriate.

Treatment of isolated organisms

The treatment of organisms identified from lower airway samples is not part of the study protocol. All results will be available for the local clinical team who will follow local guidance about the treatment.

Confidential: For Review Only

Microbiology Samples

All microbiology samples (FB-BAL, cough swabs and induced sputum samples) will be handled and stored in-line with the UK Standards for Microbiology Investigations (UK SMI).

Public and Patient Involvement

CLASSIC PBB was developed to address the concerns of families related to the burden associated with FB-BAL. We undertook detailed interviews with the parents of 25 children with PBB who have undergone FB-BAL. They identified significant burden related to anxiety about the procedure, the need to take time off school / work and financial implications.[10] These results highlighted the need to look for a non-invasive alternative to FB-BAL for children with PBB and have helped us decide on the topics for the parental assessment of tolerability. Three parent contributors helped develop the study methodology which was also presented to the Alder Hey Young Persons Advisory Group (YPAG).

One of the parent contributors (EH) is PPIE lead for the study, a co-applicant on the funding application and co-author on this paper. She has helped develop the PIS and consent / assent forms as well as being a member of the Trial Steering Committee where she will continue to be an advocate of parents and children. Once the study is complete, she will help in writing the plain English summary of the results.

Statistical Data Analysis

<u>Sample Size Calculation</u>

The sample size was calculated using the McNemar's test[16] for paired proportions, informed by the comparison of pathogen yield from paired induced sputum and six-lobe FB-BAL samples in the CF-SPIT study.[15] Assuming a proportion of discordant pairs of 0.37 we require a sample size of 110 to detect a ratio of discordant proportions (identification of an organism[s] on FB-BAL not isolated on induced sputum vs identification of organism[s] on induced sputum not identified on FB-BAL) of 3, with 90% power and 0.05 significance. If we assume the same induced sputum success rate as CF-SPIT (84%) we need a final sample size of 131. The sample size required to detect a difference between cough swab and FB-BAL is smaller due to a higher proportion of discordant pairs.

<u>Primary Outcome Analysis</u>

The organisms isolated from each participants cough swab will be compared to those isolated from their FB-BAL samples to identify concordance / discordance. The pathogen yield is concordant if the same organism(s) are identified in both samples or no organisms are identified in either. If the same organisms are not identified in both samples the pathogen yield is discordant. Discordance will be classified as CS^{+}/FB-BAL^{-} when an organism is isolated on cough swab but not on FB-BAL or CS^{-}/FB-BAL^{+} when an organism is isolated on FB-BAL but not on cough swab. This will be repeated with the FB-BAL organisms limited to those from a single lobe (RML) and two lobes (RML & LLi) to allow comparison of pathogen yield if a more limited FB-BAL sampling methodology had been used. The same analysis will be undertaken to identify concordance / discordance between the induced sputum and the FB-BAL samples.

https://mc.manuscriptcentral.com/bmjpo

Confidential: For Review Only

We will use conditional logistic regression analyses to estimate the odds ratio of discordant proportions for the paired cough swabs and FB-BAL samples (identification of an organism[s] on FB-BAL not isolated on cough swab versus identification of organism[s] on cough swab not identified on FB-BAL). This will be analysed separately using FB-BAL results from one, two and six lobes. A similar analysis will be undertaken for the paired induced sputum and FB-BAL samples.

Secondary Outcomes Analysis

1. We will estimate the sensitivity for each sampling technique against a combined gold standard consisting of all pathogens isolated from the triplicate samples (cough swab, sputum induction and six-lobe FB-BAL). A positive outcome will be defined as the ability of a single technique to identify all pathogens from the combined gold standard. This will enable us to quantify the ability of each technique to correctly detect all lower airway pathogens in any given patient. Two-sided 95% score confidence intervals for sensitivity will be estimated.
2. We will use a logistic regression analysis to assess the effect of age on pathogen positivity and the success rate of sputum induction.
3. The Likert score for tolerability will be summarised using a frequency table stratified for age.

Study Oversight and Monitoring

Trial Management Group (TMG)

The TMG will be responsible for the day-to-day management of the study. It will consist of the Chief Investigator (CI), Principal Investigators (PIs), Statistician, Clinical Trial Manager, Data Manager, Data Co-ordinator, Quality Assurance Manager and Microbiology Lead.

Trial Steering Committee (TSC)

The TSC will provide supervision for the trial / study on behalf of the Sponsor and Funder. It will also provide expert advice independent of the CI and the Sponsor. The TSC will consist of an independent chair, two independent paediatric respiratory consultants, an independent statistician, the PPIE Lead and the CI.

Study Monitoring

Monitoring will be undertaken in accordance with the Study Monitoring Plan. The monitor may review processes related to participant enrolment, eligibility, consent and adherence. They will also assess policies to protect participants, including reporting of harm and completeness, accuracy, and timeliness of data collection. Monitoring may be carried out remotely by exploring the study dataset or performing site visits.

Data Management

Data will be collected in accordance with the Data Management Plan using CRFs. The completed CRFs will be returned to the Data Coordinator and inputted onto the REDCap database. Data quality checks will be conducted periodically to ensure accuracy of the primary and secondary outcomes.

Confidential: For Review Only

Funding, Ethical Approval and Trial Registration

Funding

CLASSIC PBB has been funded by an NIHR Research for Patient Benefit Grant (NIHR202272)

REC Approval

CLASSIC PBB was approved by London Central Research Ethics Committee (IRAS Project ID: 229341).

Clinical Trial Registration

CLASSIC PBB has been registered on the ISRCTN Registry (ISRCTN79883982)

Study Progress

This 24-month trial opened on 01/10/2021. Recruitment started on 01/01/2022 and will be open for 17 months.

Discussion

CLASSIC PBB will compare the pathogen yield from different lower airway sampling strategies in children with PBB. If either cough swab or induced sputum are shown to be useful alternatives to FB-BAL, it will enable more children with PBB to have the causative pathogen identified and therefore be prescribed targeted antibiotics. It would also reduce the need for FB-BAL in this condition which would be beneficial to children and their families due to the associated burden.

Cough swabs and induced sputum samples are not used routinely in children with PBB. They are, however, widely used in children with other respiratory illnesses who are unable to expectorate sputum. At UK paediatric cystic fibrosis (CF) Centres, a cough swab is obtained at each out-patient appointment (2-3 monthly) as part of routine microbiological surveillance.[17] They are easy to perform and well tolerated. The CF-SPIT study compared pathogen yield from cough swab, induced sputum and FB-BAL samples in children aged 6 months to 18 years with CF. They reported 69% of pathogens isolated at FB-BAL had been identified in cough swabs over the previous 12 months but a single cough swab was not representative of lower airway microbiology in CF. This supports the findings of a previous study which found the sensitivity of a single cough swabs to be 44% when compared to FB-BAL samples.[18]

Induced sputum has been shown to be a simple, cost effective, well tolerated and repeatable method of sampling the lower airway in children with CF.[15,19] In the CF-SPIT study, the pathogen yield of 41 paired induced sputum and FB-BAL samples was compared.[15] Of the 41 paired samples, 28 (68%) had a positive growth from at least one of the samples with 39 pathogens isolated in total. Induced sputum identified 27 (69%) pathogens, single-lobe FB-BAL identified 22 (56%), two-lobe FB-BAL 28 (72%) and six-lobe FB-BAL identified 33 (85%). Importantly, some pathogens were isolated from induced sputum and not FB-BAL reflecting the relative inability of FB-BAL to sample the larger airways. The authors of the CF-SPIT concluded that in children with CF, sputum induction is a credible surrogate for bronchoalveolar lavage and a substantial number of FB-BALs could be avoided if sputum induction is done first and pathogens appropriately treated.[15]

https://mc.manuscriptcentral.com/bmjpo

Confidential: For Review Only

Although induced sputum sounds unpleasant, the success and tolerability rates in published cohorts is high. The three largest cohorts (CF-SPIT[15] and two studies of children with suspected TB[20,21]) report 1,099 attempts at induced sputum in 523 children aged 1 month to 16 years. A sputum sample was successfully obtained in 94% of procedures. Objective tolerance of the sputum induction procedure is good with CF-SPIT reporting no significant effects on respiratory rate, heart rate, or FEV_1%. Subjective tolerance was also good. Likert scales rated tolerance high, with mean parent or patient scores of 8·55 (SD 1·65) and physiotherapist scores of 9·09 (1·76). During the 200 induced sputum reported in CF-SPIT, there was a low incidence of side-effects: 17 (9%) became upset, of which four (2%) could not complete the procedure; six (3%) had mild wheeze, of which two (1%) could not complete the procedure; three (2%) patients vomited during oropharyngeal suction and one (<1%) became transiently dizzy.

Whilst induced sputum has been shown to be a useful alternative to FB-BAL in children with CF and TB it cannot be assumed this is the case for PBB. Children with PBB are younger than those with CF and the respiratory pathology is less suppurative. These factors potentially affect the likelihood of successfully obtaining samples.

Confidential: For Review Only

References

1. Brodlie M, Graham C, McKean MC. Childhood cough. BMJ 2012;344:e1177.

2. Marchant JM, Masters IB, Taylor SM, Cox NC, Seymour GJ, Chang AB. Evaluation and outcome of young children with chronic cough. Chest 2006;129(5):1132–41.

3. Craven V, Everard ML. Protracted bacterial bronchitis: reinventing an old disease. Arch Dis Child 2013;98(1):72–6.

4. Chang AB, Robertson CF, Van Asperen PP, et al. A multicenter study on chronic cough in children : burden and etiologies based on a standardized management pathway. Chest 2012;142(4):943–50.

5. Newcombe PA, Sheffield JK, Chang AB. Parent cough-specific quality of life: development and validation of a short form. J Allergy Clin Immunol 2013;131(4):1069–74.

6. Chang AB, Redding GJ, Everard ML. Chronic wet cough: Protracted bronchitis, chronic suppurative lung disease and bronchiectasis. Pediatr Pulmonol 2008;43(6):519–31.

7. Wurzel DF, Marchant JM, Yerkovich ST, et al. Protracted Bacterial Bronchitis in Children: Natural History and Risk Factors for Bronchiectasis. Chest 2016;150(5):1101–8.

8. Chang AB, Bush A, Grimwood K. Bronchiectasis in children: diagnosis and treatment. Lancet 2018;392(10150):866–79.

9. Narang R, Bakewell K, Peach J, et al. Bacterial distribution in the lungs of children with protracted bacterial bronchitis. PLoS ONE 2014;9(9):e108523.

10. Swift JA, Carroll WD, Gilchrist FJ. Single-centre survey of parents regarding the hidden burden of paediatric flexible bronchoscopy. BMJ Paediatr Open 2021;5(1):e000991.

11. Chang AB, Upham JW, Masters IB, et al. Protracted bacterial bronchitis: The last decade and the road ahead. Pediatr Pulmonol 2016;51(3):225–42.

12. Gilchrist FJ. An approach to the child with a wet cough. Paediatr Respir Rev 2019;31:75–81.

13. Marchant J, Masters IB, Champion A, Petsky H, Chang AB. Randomised controlled trial of amoxycillin clavulanate in children with chronic wet cough. Thorax 2012;67(8):689–93.

14. Pritchard MG, Lenney W, Gilchrist FJ. Outcomes in children with protracted bacterial bronchitis confirmed by bronchoscopy. Arch Dis Child 2015;100(1):112.

15. Ronchetti K, Tame J-D, Paisey C, et al. The CF-Sputum Induction Trial (CF-SpIT) to assess lower airway bacterial sampling in young children with cystic fibrosis: a prospective internally controlled interventional trial. Lancet Respir Med 2018;6(6):461–71.

16. Julious SA, Campbell MJ, Altman DG. Estimating sample sizes for continuous, binary, and ordinal outcomes in paired comparisons: practical hints. J Biopharm Stat 1999;9(2):241–51.

17. Forton JT. Detecting respiratory infection in children with cystic fibrosis: Cough swab, sputum induction or bronchoalveolar lavage. Paediatr Respir Rev 2019;31:28–31.

Confidential: For Review Only

18. Rosenfeld M, Emerson J, Accurso F, et al. Diagnostic accuracy of oropharyngeal cultures in infants and young children with cystic fibrosis. Pediatr Pulmonol 1999;28(5):321–8.

19. Al-Saleh S, Dell SD, Grasemann H, et al. Sputum induction in routine clinical care of children with cystic fibrosis. J Pediatr 2010;157(6):1006-1011.e1.

20. Zar HJ, Tannenbaum E, Apolles P, Roux P, Hanslo D, Hussey G. Sputum induction for the diagnosis of pulmonary tuberculosis in infants and young children in an urban setting in South Africa. Arch Dis Child 2000;82(4):305–8.

21. Zar HJ, Hanslo D, Apolles P, Swingler G, Hussey G. Induced sputum versus gastric lavage for microbiological confirmation of pulmonary tuberculosis in infants and young children: a prospective study. Lancet 2005;365(9454):130–4.

Confidential: For Review Only

From: Gilchrist, Francis (RJE) UHNM
Sent: 25 October 2022 10:05
To: onbehalfof@manuscriptcentral.com
Subject: bmjpo-2022-001722

Imti

Further to your email on 22nd October please find a response from Scott Thomson (NIHR RfPB Program Manager) confirming that the protocol was peer reviewed as part of the funding application. Please let me know if you need any more information.

Kind regards

Fran

Dr Francis Gilchrist. MBChB, FRCPCH, PhD
Senior Lecturer and Consultant in Paediatric Respiratory Medicine
Keele University and Royal Stoke University Hospital

Begin forwarded message:

From: Scott Thomson <scott.thomson@nihr.ac.uk>
Date: 25 October 2022 at 09:46:04 BST
To: "Gilchrist, Francis (RJE) UHNM" <Francis.Gilchrist@uhnm.nhs.uk>
Subject: Re: A favour!

This message was received from **outside** of UHNM. **STOP**. Were you expecting this email? Does it look genuine? **THINK**. Before you CLICK on links or OPEN any attachments

Hi Fran,

Re: NIHR202272 - Comparison of Lower Airway Sampling Strategies In Children with PBB (CLASSIC PBB).

I'm well thanks, I hope you are too.

I can confirm that the application and the research plan of the above project were reviewed by an expert committee and by external peer-reviewers as part of a funding decision process.

I would hope this email would suffice, but if it needs to be on headed paper, or if any additional clarifications are required, please let me know.

Best wishes,

Scott

--
Dr. Scott Thomson
Programme Manager | Research Programmes: RfPB | NIHR Central Commissioning Facility (CCF)
t. +442036927971 | **e.** scott.thomson@nihr.ac.uk

NIHR | National Institute for Health and Care Research

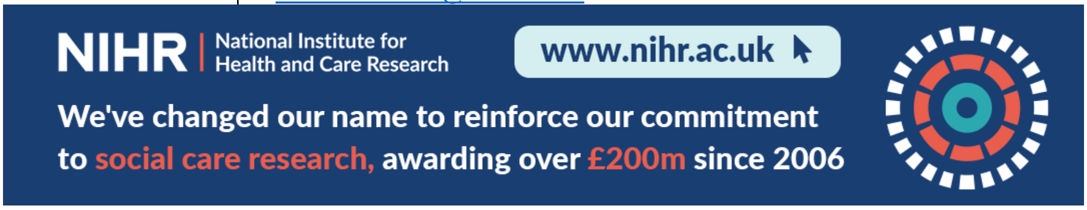

Confidential: For Review Only

Follow us: OfficialNIHR | @NIHRresearch | NIHR | NIHRtv | nihr.ac.uk

Confidential information may be contained in this message.If you are not the intended recipient, any reading, printing, storage, disclosure, copying or any other action taken in respect of this e-mail is prohibited and may be unlawful. If you are not the intended recipient, please notify the sender immediately by using the reply function and then permanently delete what you have received.

On Sat, 22 Oct 2022 at 13:34, Gilchrist, Francis (RJE) UHNM <Francis.Gilchrist@uhnm.nhs.uk> wrote:

Hi Scott

I hope you are well. I am email in the hope of a small favour.

I am have just submitted the protocol for the CLASSIC PBB Study (NIHR202272) for publication with BMJ Paediatrics Open. As you will see from the email below, they have asked me to confirm if NIHR RfPB peer reviewed the application as if you did it saves them having to do this. I would be really grateful if you could email me a short letter on headed confirming this.

Many thanks

Fran

Dr Francis J Gilchrist

MBChB(Hons), FRCPCH, PhD
Senior Lecturer and Consultant in Paediatric Respiratory Medicine

---

From: BMJ Paediatrics Open <onbehalfof@manuscriptcentral.com>
Sent: 22 October 2022 11:56
To: Gilchrist, Francis (RJE) UHNM
Subject: [BULK] bmjpo-2022-001722 - Editor email

This message was received from outside of UHNM. STOP. Were you expecting this email? Does it look genuine? THINK. Before you CLICK on links or OPEN any attachments

22-Oct-2022

bmjpo-2022-001722 - The protocol for CLASSIC PBB: Comparison of Lower Airway Sampling Strategies in Children with Protracted Bacterial Bronchitis

Dear Francis,
Can you confirm that NIHR Research for Patient Benefit peer reveiwed your protocol? If so, it avoids the need for peer review and goes straight to editorial review. We will however need a copy of the letter stating that it was peer reviewed

Kind regards,

Prof. Imti Choonara
BMJ Paediatrics Open

Confidential: For Review Only

NHS
Health Research Authority

London - Central Research Ethics Committee
3rd Floor, Barlow House
4 Minshull Street
Manchester
M1 3DZ

**<u>Please note</u>: This is an acknowledgement letter from the REC only and does not allow you to start your study at NHS sites in England until you receive HRA Approval.**

19 October 2021

Dr Francis Gilchrist
Senior Lecturer and Consultant in Paediatric Respiratory Medicine
University Hospitals of North Midlands NHS Trust
Royal Stoke University Hospital (UHNM)
Newcastle Road
Staffordshire
ST4 6QG

Dear Dr Gilchrist,

**Study title:**	**A multi-centre, prospective, cross-sectional cohort study comparing the microbiology yields of cough swab and induced sputum samples with that of bronchoalveolar lavage samples obtained during flexible bronchoscopy, in children with protracted bacterial bronchitis.**
**REC reference:**	**21/LO/0689**
**Protocol number:**	**3098**
**IRAS project ID:**	**299341**

Thank you for your letter of 13 October 2021. I can confirm the REC has received the documents listed below and that these comply with the approval conditions detailed in our letter dated 05 October 2021

Documents received

The documents received were as follows:

Document	*Version*	*Date*
Covering letter on headed paper [Cover Letter]		12 October 2021
Other [IRAS Form Text Update]	1.0	12 October 2021
Participant consent form [UoB ICF]	1.1	12 October 2021
Participant consent form [ICF]	1.1	12 October 2021
Participant information sheet (PIS) [Parent PIS]	1.1	12 October 2021

Confidential: For Review Only

NHS
Health Research Authority

Participant information sheet (PIS) [Age 6-10 PIS]	1.1	12 October 2021
Participant information sheet (PIS) [UoB PIS]	1.1	12 October 2021
Research protocol or project proposal [Protocol]	1.1	12 October 2021
Summary, synopsis or diagram (flowchart) of protocol in non technical language [Summary Sheet]	1.1	12 October 2021

Approved documents

The final list of approved documentation for the study is therefore as follows:

Document	*Version*	*Date*
Covering letter on headed paper [Cover Letter]		25 August 2021
Covering letter on headed paper [Cover Letter]		12 October 2021
GP/consultant information sheets or letters [GP Letter]	1.0	24 August 2021
IRAS Application Form [IRAS_Form_26082021]		26 August 2021
Letter from funder [Funder Letter]		18 August 2021
Letter from sponsor [Sponsor Letter]		25 August 2021
Non-validated questionnaire [Questionnaire]	1.0	24 August 2021
Other [IRAS Form Text Update]	1.0	12 October 2021
Participant consent form [Assent Form]	1.0	24 August 2021
Participant consent form [UoB ICF]	1.1	12 October 2021
Participant consent form [ICF]	1.1	12 October 2021
Participant information sheet (PIS) [Age 1-5 PIS]	1.0	24 August 2021
Participant information sheet (PIS) [Age 6-10 PIS]	1.1	12 October 2021
Participant information sheet (PIS) [UoB PIS]	1.1	12 October 2021
Participant information sheet (PIS) [Parent PIS]	1.1	12 October 2021
Research protocol or project proposal [Protocol]	1.1	12 October 2021
Summary CV for Chief Investigator (CI) [CI CV]		09 September 2020
Summary, synopsis or diagram (flowchart) of protocol in non technical language [Summary Sheet]	1.1	12 October 2021

You should ensure that the sponsor has a copy of the final documentation for the study. It is the sponsor's responsibility to ensure that the documentation is made available to R&D offices at all participating sites.

IRAS Project ID: 299341 Please quote this number on all correspondence

Yours sincerely,

Mr Liam Hutchinson
Approvals Officer

Confidential: For Review Only

NHS
Health Research Authority

London - Central Research Ethics Committee
3rd Floor, Barlow House
4 Minshull Street
Manchester
M1 3DZ

<u>Please note</u>: This is an acknowledgement letter from the REC only and does not allow you to start your study at NHS sites in England until you receive HRA Approval.

19 October 2021

Dr Francis Gilchrist
Senior Lecturer and Consultant in Paediatric Respiratory Medicine
University Hospitals of North Midlands NHS Trust
Royal Stoke University Hospital (UHNM)
Newcastle Road
Staffordshire
ST4 6QG

Dear Dr Gilchrist,

**Study title:**	**A multi-centre, prospective, cross-sectional cohort study comparing the microbiology yields of cough swab and induced sputum samples with that of bronchoalveolar lavage samples obtained during flexible bronchoscopy, in children with protracted bacterial bronchitis.**
**REC reference:**	**21/LO/0689**
**Protocol number:**	**3098**
**IRAS project ID:**	**299341**

Thank you for your letter of 13 October 2021. I can confirm the REC has received the documents listed below and that these comply with the approval conditions detailed in our letter dated 05 October 2021

Documents received

The documents received were as follows:

Document	*Version*	*Date*
Covering letter on headed paper [Cover Letter]		12 October 2021
Other [IRAS Form Text Update]	1.0	12 October 2021
Participant consent form [UoB ICF]	1.1	12 October 2021
Participant consent form [ICF]	1.1	12 October 2021
Participant information sheet (PIS) [Parent PIS]	1.1	12 October 2021

Confidential: For Review Only

NHS
Health Research Authority

Participant information sheet (PIS) [Age 6-10 PIS]	1.1	12 October 2021
Participant information sheet (PIS) [UoB PIS]	1.1	12 October 2021
Research protocol or project proposal [Protocol]	1.1	12 October 2021
Summary, synopsis or diagram (flowchart) of protocol in non technical language [Summary Sheet]	1.1	12 October 2021

Approved documents

The final list of approved documentation for the study is therefore as follows:

Document	*Version*	*Date*
Covering letter on headed paper [Cover Letter]		25 August 2021
Covering letter on headed paper [Cover Letter]		12 October 2021
GP/consultant information sheets or letters [GP Letter]	1.0	24 August 2021
IRAS Application Form [IRAS_Form_26082021]		26 August 2021
Letter from funder [Funder Letter]		18 August 2021
Letter from sponsor [Sponsor Letter]		25 August 2021
Non-validated questionnaire [Questionnaire]	1.0	24 August 2021
Other [IRAS Form Text Update]	1.0	12 October 2021
Participant consent form [Assent Form]	1.0	24 August 2021
Participant consent form [UoB ICF]	1.1	12 October 2021
Participant consent form [ICF]	1.1	12 October 2021
Participant information sheet (PIS) [Age 1-5 PIS]	1.0	24 August 2021
Participant information sheet (PIS) [Age 6-10 PIS]	1.1	12 October 2021
Participant information sheet (PIS) [UoB PIS]	1.1	12 October 2021
Participant information sheet (PIS) [Parent PIS]	1.1	12 October 2021
Research protocol or project proposal [Protocol]	1.1	12 October 2021
Summary CV for Chief Investigator (CI) [CI CV]		09 September 2020
Summary, synopsis or diagram (flowchart) of protocol in non technical language [Summary Sheet]	1.1	12 October 2021

You should ensure that the sponsor has a copy of the final documentation for the study. It is the sponsor's responsibility to ensure that the documentation is made available to R&D offices at all participating sites.

**IRAS Project ID: 299341**	**Please quote this number on all correspondence**

Yours sincerely,

Mr Liam Hutchinson
Approvals Officer

Confidential: For Review Only
