# Supplementary material for: Protocol for CLASSIC PBB: comparison of lower airway sampling strategies in children with protracted bacterial bronchitis
Source: BMJ Paediatr Open. 2022 Nov 21;6(1):e001722. doi: 10.1136/bmjpo-2022-001722 (PMC9680323; doi:10.1136/bmjpo-2022-001722)
Supplement: Reviewer comments [file bmjpo-2022-001722.reviewer_comments.pdf]

PEER REVIEW HISTORY

BMJ Paediatrics Open publishes all reviews undertaken for accepted manuscripts. Reviewers are asked to complete a checklist review form and are provided with free text boxes to elaborate on their assessment. These free text comments are reproduced below.

ARTICLE DETAILS

**TITLE (PROVISIONAL)**	The protocol for CLASSIC PBB: Comparison of Lower Airway Sampling Strategies in Children with Protracted Bacterial Bronchitis
**AUTHORS**	Gilchrist, Francis J<br>Aspey, Mathew<br>Bowler, Robert<br>Brodlie, Malcolm<br>Desai, Seema<br>Harris, Caroline<br>Hinton, Emily<br>Kulkarni, Hemant<br>Ogbolosingha, Aviva<br>Sinha, Ian<br>Solis-Trapala, Ivonne<br>Stock, Joanne<br>Carroll, William D

VERSION 1 – AUTHOR RESPONSE

Many thanks for the comments on this protocol. I have made the amendment to the Discussion as suggested and uploaded the REC Approval and the confirmation from NIHR RfPB that the protocol underwent peer review as part of the funding process.
